# State Severity Assessment in Patients with Obstructive Jaundice using PMGMU2018h Scale Adapted to the Russian Medical and Economic Standards

**DOI:** 10.17691/stm2020.12.3.10

**Published:** 2020-06-28

**Authors:** А.N. Scherbyuk, S.S. Dydykin, P.А. Ivanov, V.I. Laptina, V.М. Маnuylov, М.V. Nelipa, А.N. Levitskaya, K.N. Levitskaya, P.V. Kruchko, K.А. Zhandarov

**Affiliations:** Professor, Department of Operative Surgery and Topographic Anatomy; I.M. Sechenov First Moscow State Medical University (Sechenov University), 8/2 Malaya Trubetskaya St., Moscow, 119991, Russia; Head of the Department of Operative Surgery and Topographic Anatomy; I.M. Sechenov First Moscow State Medical University (Sechenov University), 8/2 Malaya Trubetskaya St., Moscow, 119991, Russia; Professor, Research Adviser; N.V. Sklifosovsky Research Institute of Emergency Care, 3 Bolshaya Sukharevskaya Square, Moscow, 129090, Russia; Assistant, Department of Operative Surgery and Topographic Anatomy; I.M. Sechenov First Moscow State Medical University (Sechenov University), 8/2 Malaya Trubetskaya St., Moscow, 119991, Russia; Chief Doctor; Pushkino District Hospital named after Prof. V.N. Rozanova, 35 Aviatsionnaya St., Pushkino, Moscow Region, 141206, Russia; Associate Professor, Department of Operative Surgery and Topographic Anatomy; I.M. Sechenov First Moscow State Medical University (Sechenov University), 8/2 Malaya Trubetskaya St., Moscow, 119991, Russia; Surgeon; Pushkino District Hospital named after Prof. V.N. Rozanova, 35 Aviatsionnaya St., Pushkino, Moscow Region, 141206, Russia; Student; I.M. Sechenov First Moscow State Medical University (Sechenov University), 8/2 Malaya Trubetskaya St., Moscow, 119991, Russia; Student; I.M. Sechenov First Moscow State Medical University (Sechenov University), 8/2 Malaya Trubetskaya St., Moscow, 119991, Russia; Assistant, Department of Operative Surgery and Topographic Anatomy; I.M. Sechenov First Moscow State Medical University (Sechenov University), 8/2 Malaya Trubetskaya St., Moscow, 119991, Russia; Associate Professor, Department of Operative Surgery and Topographic Anatomy; I.M. Sechenov First Moscow State Medical University (Sechenov University), 8/2 Malaya Trubetskaya St., Moscow, 119991, Russia

**Keywords:** formula of the severity degree for patients with jaundice, indicators of obstructive jaundice, PMGMU2018h scale, APACHE II, SAPS, SOFA, MODS.

## Abstract

**The aim of the study** was to assess the effectivity of PMGMU2018h scale for evaluation of the state severity degree of patients suffering from obstructive jaundice relative to other common assessment scales.

**Materials and Methods.:**

Thirty physical parameters have been studied and compared according to different assessment scales in each of 258 patients with obstructive jaundice treated in three medical settings.

**Results.:**

The main drawback of the examined scales is the necessity to use the parameters for calculations not included in the medical and economic standards of the Russian Federation. This feature makes these scales unsuitable for making decisions on the tactics of managing a concrete patient in the hospitals of the Russian Federation. The scale developed by us for the assessment of the state severity of patients suffering from obstructive jaundice is completely devoid of subjectivism, does not depend on a surgeon’s qualifications, and possesses high specificity to the given disease.

## Introduction

An accurate assessment of the state severity of patients with obstructive jaundice at the time of their hospitalization and during the entire stay at the surgical unit is extremely important for identification of persons who need intensive care since their admission to define the measures for arresting clinical manifestations of the disease, selecting the subsequent treatment tactics, and evaluating the disease prognosis. Insufficient severity assessment and inadequate treatment may result in heavy complications up to the fatal outcome, while in case of overestimation, the unnecessary diagnostic and treatment procedures are most likely to be administered [1–3].

A complex assessment of the patient’s state severity is based on the clinical data obtained on admission to the surgical setting and their correlation with systemic organ failures is also taken into consideration. This approach allows one to differentiate the course of the disease as light, medium, or severe [2, 4—7]. In his practical work, a surgeon often evaluates the patient’s state relying on his own experience but his opinion is sufficiently subjective and does not always reflect a real picture. An objective assessment of the severity state by various specialists poses certain difficulties [3, 8, 9].

Presently, numerous standardized scales are used to objectively assess the patient’s state severity and predict the disease course. The most widely adopted scales such as APACHE II, SAPS, SOFA, MODS have been chosen by us for the study [1, 2, 5, 10–12]. However, these scales are not commonly used in the Russian medical settings as they apply indicators which are not included into the medical and economic standards of the Russian Federation (MES RF). This inspired us to develop our own scale for assessing the state severity of patients with obstructive jaundice which would be based on the MES RF parameters. On the basis of the conducted investigations, a PMGMU2018h scale [13] has been worked out which satisfies all the needs of our clinics.

**The aim of the study** was to compare the effectivity of the known scales for the severity assessment which are employed for patients with obstructive jaundice and the scale developed by us based on the severity degree coefficient which is determined by objective and quantitative parameters available to any practicing physician using a standard office program MS Excel.

In the course of the investigation, the following tasks were to be solved:

1. to analyze the principles of calculation of the severity assessment in each of the selected scales;
2. to study the necessary clinical and laboratory indicators which are used in them;
3. to track the changes in the parameters of an individual patient in dynamics;
4. to evaluate the severity degree calculated by different scales for a specific patient in dynamics;
5. to select the scales which meet the requirements of MES RF related to obstructive jaundice.

## Materials and Methods

Thirty physical and laboratory indicators have been studied in 258 patients with obstructive jaundice treated in three medical settings: N.V. Sklifosovsky Research Institute of Emergency Care, Clinical Center of I.M. Sechenov First Moscow State Medical University (Sechenov University), and Pushkino District Hospital named after Prof. V.N. Rozanova from 1996 to 2014.

### Statistical data processing.

To quantify the severity degree of a patient at any time with the help of the multivariate linear regression analysis using a universal statistical program package StatSoft Statistica 10.0 for MS Exel, a mathematical relation has been established between the clinical quantitative parameters and the probability of a lethal outcome or recovery. The principle and methodology of calculations have been reported by the authors in detail in their previous works [13, 14]. As a result, 9 factors significant for the quantitative definition of the patient’s state severity have been determined.

## Results and Discussion

Due to the subjective factor in determining the state severity by a surgeon, there are always doubts in the identity of this definition not only by the specialists of various clinics but physicians within the same unit as well. However, the objective assessment of the patient’s state severity in a surgical pathology of the abdominal cavity and determination of the unfavorable outcome probability present some difficulties.

One of the most common integral systems for severity evaluation is APACHE II which assesses acute physiological disorders and chronic health conditions. A distinct feature of this scale is that the estimates which use the specific parameters of organ systems dysfunction are limited by the diseases of these systems, whereas the evaluation of the systems which might provide wider information about a patient’s state requires an extensive invasive monitoring. A drawback of the given scale is the possibility to employ it only for the seriously ill patients in the intensive care units for fear of overestimating the severity degree in other patients [1, 12, 15, 16].

The next to appear was a no less significant scale SAPS, which was based on the simplified APACHE II. Some evaluation parameters were removed and the most available and easily measured were preserved: there was no need to record and calculate the average AP, the parameters of blood gas content and blood creatinine concentration were excluded; “corrections” for comorbid diseases were eliminated. This scale is non-applicable as a probable tool of lethality prediction in a specific patient as its use is restricted by the lethality prediction in stratified groups of patients without taking into account the selected “main” diagnosis [1, 12, 17, 18].

The next scale to be considered is MODS. For this scale, optimal values of variables for each of the six vital systems (central nervous system, cardiovascular and respiratory systems, functions of the kidneys, liver, and hemocoagulation system) were defined. Besides, great attention was paid to the Glasgow coma scale. MODS is utilized to assess complications rather than the risk of lethal outcome like the previous scales. It may be suitable for dynamic patient observation and evaluation of a dysfunction/failure degree of separate systems and organs [1, 19].

An integral SOFA system was developed on the basis of MODS. This scale also uses six main parameters and the same variables excluding cardiovascular system. Its insufficiency was defined by a different parameter (which appeared to be more important in the assessment of multiple organ failure and may serve as an indicator of the efficacy of the conducted treatment in some diseases). This scale was designed for a fast scoring and description of a number of complications and treatment rather than for the prediction of the disease outcome [1, 12, 15, 17].

All considered scales have some disadvantages in common: insufficient prognostic capability of an outcome for a specific patient but a relatively exact prognosis of the lethal outcome probability for a group of patients, low sensitivity at a sufficiently high specificity. This allows for a more or less accurate prediction of a lethal outcome probability but makes the evaluation of the patient’s state in dynamics difficult, which is critical for the practicing physician. We think that it is the probability of the lethal outcome defined quantitatively (by a number) that serves as an indicator of the patient’s severity degree. The higher the probability of the lethal outcome, the higher the severity degree, and vice versa, the lower the probability, the lower the patient’s severity degree. This approach to the application of the prognostic techniques makes the scales suitable for dynamic control of the patient’s condition and making decisions on the treatment tactics for each concrete patient [4, 5, 10, 20].

A PMGMU2018h scale was developed to assess the state severity of patients with obstructive jaundice at any time and, consequently, to assess the efficacy of the treatment. The state severity is defined by calculations using the original mathematical formula developed by the authors where data obtained during physical examination and laboratory findings are employed.

As mentioned above, the methodology of calculating statistical dependence underlying the developed formula has been previously described by the authors in their works [13, 14]. The formula integrates 9 significant indicators: jaundice duration in days, blood bilirubin, body temperature, blood leukocytosis, heart rate, patient’s age, blood creatinine, blood lymphocytes, respiration rate.

As a result of the calculation using a multivariate linear regression analysis and the subsequent expert appraisal of the data, a relation adequately reflecting the severity degree of the patient’s state at a definite time has been found. The detected mathematical dependence is presented by the following formula:

*G* = [0.002 (*d* · *b*) + 1.2 (*t* – 36.6) + 0.001 (*Hr* · *a*) + 0.322 (*L*) + + 0.22 (16 – *Lym*) + 0.0085 (*Cr* – 60) + 0.165 (*Rr* – 20)] – 6.0,

where *G* is the severity degree; *d* — disease duration (days); *b* — blood bilirubin (μmol/L); *t* — body temperature (°С); *Hr* — heart rate per minute; *a* — patient’s age (years); *L* — blood leukocytes in the SI units (109/L); *Lym* — blood lymphocytes (%) in the clinical blood test; *Cr* — blood creatinine in the SI units (μmol/L); *Rr* — respiratory rate per minute.

This formula is available in the MS Excel program minimizing the labor efforts of the medical personnel for calculations (Table 1).

**Table 1:** Calculation of patient’s state severity degree using PMGMU2018h scale

Indicators	Observation day		
	1^st^	4^th^	10^th^
Patient’s age — *a* (years)	65	65	65
Disease duration — *d* (days)	1	4	10
Bilirubin — *b* (μmol/L)	190	190	100
Body temperature — *t* (°С)	37.3	38	37
Blood leukocytes — *L* (109/L)	12**·**10^9^	13.5**·**10^9^	9**·**10^9^
Heart rate per min — *Hr*	100	95	90
Blood creatinine — *Cr* (μmol/L)	120	105	100
Blood lymphocytes — *Lym* (%)	18	15	21
Respiratory rate per min — *Rr*	21	20	18
Severity degree — *G*	6.7	8.3	4.9

The severity degree value (*G*) was determined for a definite patient by the given formula. To facilitate the calculation of the severity degree of a patient with obstructive jaundice, several tables were made up in the MS Excel program with the nested design equations (Tables 2–6). Data of patient K. treated in one of the mentioned clinics were used as a clinical example. All indicators necessary for computing the severity degree in all considered scales were tabulated. A separate column was introduced to indicate the inclusion of a given indicator into the obstructive jaundice-related MES RF (in bold are shown those indicators that are not included in MES).

**Table 2:** Indicators revealed on physical examination

Indicators	Observation day			Inclusion in MES
	1^st^	4^th^	10^th^	
Patient’s age (years)	65	65	65	Yes
Disease duration (days)	1	4	10	Yes
Body temperature (°С)	37.3	38	37	Yes
Rectal temperature (°С)	38.3	39	38	Yes
Systolic BP	165	155	155	Yes
Average BP [(diast. · 2 + syst.)/3]	132	125	118	Yes
**Central venous pressure**	**40**	**40**	**40**	**No**
Heart rate per min	100	95	90	Yes
Respiratory rate per min	21	20	18	Yes
Chronic disease	5 points (hepatic failure)			Yes

**Table 3:** Indicators of patient’s biochemical blood test

Indicators	Observation day			Inclusion in MES
	1^st^	4^th^	10^th^	
**Na^+^ of the blood serum** **(mmol/L)**	**152**	**150**	**149**	**No**
**К^+^ of the blood serum** **(mmol/L)**	**3.2**	**3.6**	**3.7**	**No**
Bilirubin (μmol/L)	190	190	100	Yes
Creatinine (μmol/L)	120	105	100	Yes
Blood urea (mmol/L)	8	7.3	6	Yes
Serum glucose (mmol/L)	8.1	8.3	7.6	Yes

**Table 4:** Indicators of patient’s general blood and urine tests

Indicators	Observation day			Inclusion in MES
	1^st^	4^th^	10^th^	
Hematocrit (%)	40	42	39	Yes
Leukocytes (109/L)	12**·**10^9^	13.5**·**10^9^	9**·**10^9^	Yes
Thrombocytes (109/L)	180	180	200	Yes
Blood lymphocytes (%)	18	15	21	Yes

**Table 5:** Patient’s indicators for calculation of Glasgow coma scale

Indicators	Observation day		
	1^st^	4^th^	10^th^
Glasgow scale (score)	15		
Eye opening	1 point (spontaneous)		
Motor response	1 point (obeys commands)		
Verbal reaction	1 point (oriented, maintains the conversation)		

**Table 6:** Patient’s indicators determined in the gas analyzer

Indicators	Observation day			Inclusion in MES
	1^st^	4^th^	10^th^	
**Oxygenation Pa/O_2_/FiO_2_ (mm index, Hg)**	**350**	**375**	**370**	**No**
**Oxygenation PaO_2_ (mm Hg)**	**70**	**70**	**75**	**No**
**Arterial blood (pH)**	**7.39**	**7.41**	**7.42**	**No**
**Serum НСO_3_ (mmol/L)**	**25**	**27**	**27**	**No**

Using APACHE II, SAPS, SOFA, MODS, PMGMU2018h scales, the scores were calculated on the basis of the data given in the tables. The severity degree utilizing the given scales was estimated according to the previously described techniques [1, 12, 17–19]. It is clearly seen that the PMGMU2018h scale is closer to MODS than the other scales in the assessment of this parameter. Having analyzed indicators which are determined in the medical settings in Russia in compliance with MES, we have found that all scales, except PMGMU2018h, utilize indicators which are not specified by MES.

A maximal score in each scale is taken as 100%. When the severity degree is estimated according to the original techniques [1, 12, 17–19], the score which may be obtained for one and the same patient using various scales will be different. The score of the real severity degree according to the APACHE II, SAPS, SOFA, MODS, PMGMU2018h scales will be expressed by different figures: up to 20 by SOFA, MODS, PMGMU2018h scales, up to 40 — by the APACHE II scale. But to compare the values of the severity degree obtained by different techniques, these results must be brought to a single measurement system.

Orienting in each case to a maximal possible value of the severity degree and calculating with the help of a concrete scale we expressed all found indicators in percentage of its value. The scores taken by us as maximally possible were those obtained during calculation with the substitution in the computation tables and formulas of the parameters with a maximal deviation from the norm towards the increase in the analyzed group of patients (n=258). This enabled us to compare the results of calculation in the same measuring scale.

The severity degree of a given patient calculated in percentage terms on each observation day is presented in summary Table 7.

**Table 7:** Patient’s state severity according to APACHE II, SAPS, SOFA, MODS, PMGMU2018h scales in percentage of the maximal possible value

Severity degree (%)	Observation day		
	1^st^	4^th^	10^th^
APACHE II, max=40	10	–2.5	–7.5
SAPS, max=30	30	13.3	13.3
SOFA, max=20	25	20	25
PMGMU2018h, max=20	33.5	41.5	24.5
MODS, max=20	35	35	30

It is clearly seen from the Figure that the PMGMU2018h scale is close to the most extent to MODS by its assessment of the severity degree, beside only PMGMU2018h meets MES RF. The SAPS and SOFA scales have similar dynamics but their indicators of the severity degree are insignificantly lower than those of PMGMU2018h and MODS. APACHE II significantly underestimates the severity in comparison with other scales and cannot be recommended for use in patients with obstructive jaundice.

**Figure:**
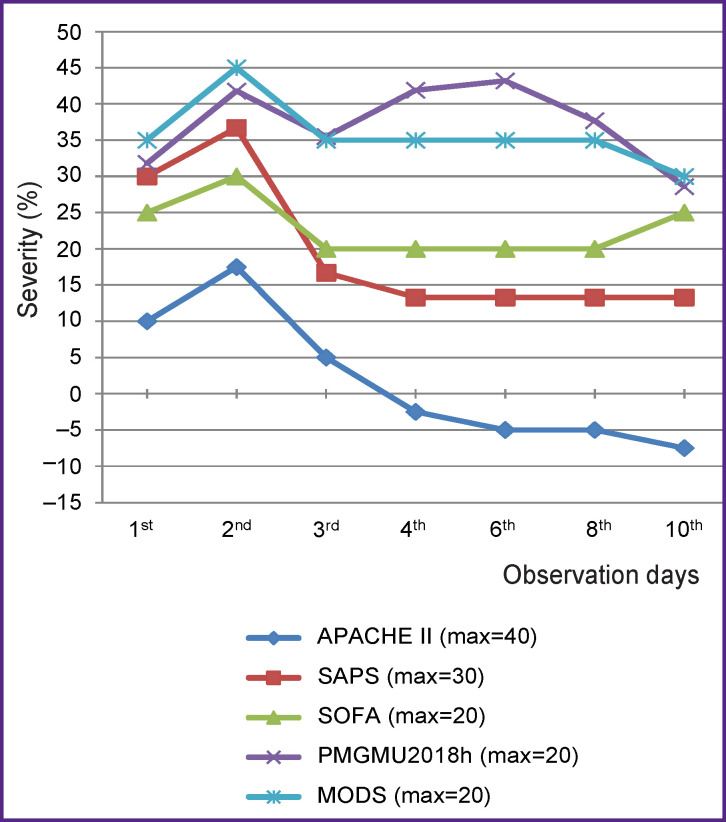
Severity of the patient’s state according to all examined scales in percentage of the maximal possible value

## Conclusion

The PMGMU2018h scale proposed by us reflects adequately the state severity degree of a patient with obstructive jaundice.

The PMGMU2018h scale provides the possibility to track minimal changes in the patient’s condition and evaluate the effect of separate treatment elements on the disease course. The proposed technique of the severity definition is completely devoid of any subjectivity and does not depend on surgeon’s qualifications.

The PMGMU2018h scale corresponds most closely to MODS. The developed scale uses only those indicators that are included in MES RF.
